# Social activity mediates locus coeruleus tangle-related cognition in older adults

**DOI:** 10.1038/s41380-024-02467-y

**Published:** 2024-02-15

**Authors:** Benjamin S. Zide, Nancy J. Donovan, Soyoung Lee, Sukriti Nag, David A. Bennett, Heidi I. L. Jacobs

**Affiliations:** 1 Division of Geriatric Psychiatry, Department of Psychiatry, Brigham and Women’s Hospital, Harvard Medical School, Boston, MA USA; 2 Department of Psychiatry, Massachusetts General Hospital, Harvard Medical School, Boston, MA USA; 3 Rush Alzheimer’s Disease Center and Department of Pathology, Rush University Medical Center, Chicago, IL USA; 4 Rush Alzheimer’s Disease Center and Department of Neurological Sciences, Rush University Medical Center, Chicago, IL USA; 5 Department of Radiology, Massachusetts General Hospital, Harvard Medical School, Boston, MA USA; 6 School for Mental Health and Neuroscience, Alzheimer Centre, Limburg, Maastricht University, Maastricht, The Netherlands

**Keywords:** Predictive markers, Diseases

## Abstract

The locus coeruleus-noradrenaline system regulates brain-wide neural activity involved in cognition and behavior. Integrity of this subcortical neuromodulatory system is proposed to be a substrate of cognitive reserve that may be strengthened by lifetime cognitive and social activity. Conversely, accumulation of tau tangles in the brainstem locus coeruleus nuclei is recently studied as a very early marker of Alzheimer’s disease (AD) pathogenesis and cognitive vulnerability, even among older adults without cognitive impairment or significant cerebral AD pathologies. This clinical-pathologic study examined whether locus coeruleus tangle density was cross-sectionally associated with lower antemortem cognitive performance and social activity among 142 cognitively unimpaired and impaired older adults and whether social activity, a putative reserve factor, mediated the association of tangle density and cognition. We found that greater locus coeruleus tangle density was associated with lower social activity for the whole sample and in the cognitively unimpaired group alone and these associations were independent of age, sex, education, depressive symptoms, and burden of cerebral amyloid and tau. The association of locus coeruleus tangle density with lower cognitive performance was partially mediated by level of social activity. These findings implicate the locus coeruleus-noradrenaline system in late-life social function and support that locus coeruleus tangle pathology is associated with lower levels of social activity, independent of cerebral AD pathologies, and specifically among older adults who are cognitively unimpaired. Early brainstem pathology may impact social function, and level of social function, in turn, influences cognition, prior to canonical stages of AD.

## Introduction

Alzheimer’s disease (AD) is biologically defined by the cerebral accumulation of amyloid-beta (Aβ) containing plaques and neurofibrillary tangles (NFT) comprised of hyperphosphorylated tau, a process that begins decades before the onset of mild cognitive impairment (MCI) and dementia [1, 2]. Earlier in life and prior to the accumulation of cerebral plaques and tangles, the small bilateral brainstem nuclei that comprise the locus coeruleus (LC) accumulate hyperphosphorylated tau as pre-tangle material [3]. Neuropathological findings support that LC tangle accumulation occurs prior to and, later, concomitant with AD pathological progression and clinical decline [4, 5]. LC tangle density has been correlated with Braak staging of NFT and, negatively, with episodic memory function in both cognitively unimpaired and cognitively impaired older adults [6]. Thus, LC tangle density may be a very early marker of biological and cognitive vulnerability during AD pathogenesis.

LC neurons are the main source of noradrenaline (NA) in the brain, projecting extensively throughout the neocortex and broadly across the neuraxis [7]. LC activity modulates and optimizes attentional and other cognitive and behavioral processes [8]. Moreover, the LC–NA system functions in concert with the hypothalamic-pituitary-adrenal (HPA) endocrine system to regulate cognitive, behavioral, and physiologic (e.g., cardiovascular, immune, and inflammatory) responses to endogenous and exogenous stresses [9, 10]. Preserved structure and function of the LC–NA system may be a form of neural reserve that supports cognitive performance in the presence of age-related and neurodegenerative brain changes [11–13]. It has also been proposed that this neural substrate of cognitive reserve may be constituted and strengthened by engagement in stimulating cognitive and social activities [13–15].

Mechanisms by which social activity potentiates cognitive function are not well-defined [15]. Social interactions may directly enhance cognition and cognitive reserve by providing mental stimulation, opportunities for verbal engagement, social cognitive activity, memory retrieval, and the potential for new learning that strengthen cortical and subcortical networks involved in these processes. Moreover, social activity could be conceptualized as a form of environmental enrichment in which arousal, novelty, and dynamic aspects of interpersonal engagement activate the LC and noradrenaline release. Animal studies have found specific increases in noradrenaline in cortical, limbic, cerebellar, and brainstem regions following experimental enrichment exposures [16, 17]. In humans, higher self-reported lifespan engagement in complex mental and physical activity has been associated with higher LC catecholamine synthesis capacity [13].

Though indirect, other perceived and actual benefits from engaging with others, such as emotional and tangible forms of social support, have been associated with lower inflammatory burden and lower cardiovascular responses to stress that can help maintain brain health and resistance to AD progression [18, 19]. While recent data have linked LC tangle pathology with poorer cognitive performance in persons with no cognitive impairment (NCI) and cognitive impairment (CI) [6], the association of LC tangle pathology with level of social activity has not been investigated to date. Furthermore, it is unknown if social activity, as a form of reserve, may partially mediate the association of LC tangle pathology with cognitive performance.

Using clinical and neuropathological data from NCI and CI older adults from the Rush Memory and Aging Project, we sought to examine: (1) the associations of LC tangle and LC neuronal densities with social activity; (2) these associations across diagnostic groups (NCI, mild cognitive impairment [MCI] and AD dementia; (3) whether these associations are independent of cerebral pathology (Aβ or NFT) and (4) whether social activity mediates the association of LC tangle pathology with cognition. We hypothesized an association of LC tangle pathology with social activity, as observed for cognition, that would be independent of cerebral pathologies among NCI and CI older adults. We predicted that social activity would partially mediate the association of LC tangle pathology and cognition.

## Methods

### Study participants

Data were derived from 142 older adults participants from the Rush Memory and Aging Project (MAP), a clinical-pathological observational study of aging and dementia that started in 1997 [20, 21]. MAP recruited older individuals from continuous care retirement communities, senior subsidized housing facilities, and individual homes around the greater Chicago metropolitan area. Participants selected for the current study had LC pathologic information and social activity measures obtained within one year, on average, before death. Participants underwent annual neuropsychological and clinical assessments and agreed to brain donation at time of death. The study was approved by an Institutional Review Board of Rush University Medical Center. All participants provided informed consent and a repository consent, which allowed their data to be shared, and they signed an Anatomic Gift Act.

### Social activity measure

A late-life social activity score was calculated from six questions which assessed how frequently participants engaged in common social activities during the past year [22]. Participants were asked how often they had: (1) gone to restaurants, sporting events or teletract, or played bingo; (2) gone on day trips or overnight trips; (3) done unpaid community/ volunteer work; (4) visited at relatives’ or friends’ houses; (5) participated in groups (such as senior center, VFW, Knights of Columbus, Rosary Society, or something similar); (6) attended church or religious services. Each question was rated on a 5-point scale according to one of the following responses: once a year or less; several times per year; several times per month; several times per week; or every day or almost every day. Item scores ranged from 1 to 5 and were averaged to yield an overall score, with a higher score indicating greater social activity. Social activity measures were used from the clinical visit closest to death (social activity) and, for secondary analyses, from the first clinical visit (first social activity).

### Cognitive function and other clinical measures

Global cognition was calculated as the mean *z* score from a battery of neuropsychological assessments testing various domains of memory (episodic, semantic, and working) as well as visuospatial ability and perceptual speed [23]. Participants received clinical diagnoses of no cognitive impairment (NCI), mild cognitive impairment (MCI) or AD dementia in the following manner. At the time of death, all available clinical data were reviewed by a neurologist with expertise in dementia, and a summary diagnostic opinion was rendered regarding the most likely clinical diagnosis. Summary diagnoses were made blinded to all post-mortem data. Case conferences including one or more neurologists and a neuropsychologist were used for consensus on selected cases [24–26]. Cognition measures were used from the visit closest to death (cognition) and, for secondary analyses, from the first clinical visit (first cognition).

Depressive symptoms were measured using a modified, 10-item version of the Center for Epidemiological Studies-Depression (CES-D) [27]. Participants were genotyped via high-throughput sequencing and then classified as carriers or non-carriers of the Apolipoprotein E (APOE) ε4 allele [28].

Physical activity was assessed using questions from the 1985 National Health Interview Survey measuring the sum of hours per week that a participant engages in the following activities: walking for exercise, gardening or yard work, calisthenics or general exercise, bicycle riding and swimming or water exercises. The number of occasions and average minutes per occasion over the last two weeks were reported, summed, and expressed as hours per week [29]. Physical activity measures were used from the visit closest to death (physical activity) and from the first clinical visit (first physical activity).

### Neuropathological measures

The brain and brainstem were removed at autopsy and fixed in 4% paraformaldehyde in 0.1 M phosphate buffer for 73 h. Two transverse blocks of fixed brainstem containing 2 levels of both the left and right LC (*n* = 4) were collected as described previously [29]. All blocks were embedded in paraffin and immunohistochemistry for tyrosine hydroxylase was used to quantify LC neuronal density, and AT8 was used to obtain LC neurofibrillary tangle (NFT) density/mm^2^ (Fig. S1 and supplementary methods for more details). To quantify the cerebral NFT, five brain regions (midfrontal, middle temporal, inferior parietal, and entorhinal cortices and CA1 sector of the hippocampus) were evaluated and converted into a summary measure (NFT/mm^2^) as described previously [30]. For the percentage area occupied by Aβ, immunoreactive plaques were quantified in eight brain regions (anterior cingulate, dorsolateral prefrontal, superior frontal, inferior temporal, entorhinal, angular/supramarginal and calcarine cortices, and the hippocampus). Values for all regions were averaged to yield a composite measure of Aβ deposition. The mean of the square root of values was used for analyses since it has better statistical properties. More details on both cerebral NFT and Aβ are provided in the supplemental data. Post-mortem interval was calculated as the time in hours between death and preservation of tissue.

### Statistical analyses

R software was utilized for the statistical analyses (R, version 4.1.2 R Foundation for Statistical Computing, Vienna, Austria). All tests were 2-tailed, and the alpha level was set as *p* < 0.05 for the analyses. Normality tests were used to evaluate assumptions of normality for each variable. Demographic and clinical variables were compared across the three diagnostic groups using analysis of variance, Tukey tests, and Fisher exact tests. As this was a hypothesis-driven hierarchical analysis, we did not correct for multiple comparisons.

In the first series of models, we examined the associations of LC tangle density with social activity, the dependent variable, adjusting for age, sex, and education. In analogous models, we examined the associations of LC neuronal density, AD neuropathological markers (Aβ, NFT), and depressive symptoms with social activity, adjusting for the same covariates. Subsequently, we evaluated the associations of LC tangle density with social activity, adjusting for the other neuropathological markers (LC neuronal density, Aβ, NFT) and depressive symptoms. Given the non-normality of the distributions and model residuals, the estimates were bootstrapped with 5000 replicates, generating 95% confidence intervals. We repeated these models using the social activity data collected at the first clinical visit (first social activity).

In the second series of models, we examined between-group and within-group differences in the association of LC tangle density with social activity among the NCI, MCI, and AD dementia groups, adjusting for age, sex, and education. First, we evaluated the interactive association of diagnostic group and LC tangle density with social activity. Second, we evaluated the association of LC tangle density with social activity using separate models for the NCI group and a CI group, which combined the MCI and AD dementia groups. Post-hoc models further adjusted for depressive symptoms, amyloid, and NFT.

Lastly, in separate models using the full sample, we evaluated the associations of LC tangle density and social activity with cognition, adjusting for age, sex, education, and depressive symptoms. To explore these interrelationships further, mediation analyses were implemented, informed by the available literature demonstrating that there is a protracted accumulation of tangles in the LC and that social activity can enhance resilience against cognitive decline [3, 31]. We tested whether social activity (close to death as well as at the first clinical visit) mediated the relationship between LC tangle density and cognition. As an alternative model, we considered cognition at the first clinical visit as the mediator of the relationship between LC tangle pathology and social activity close to death. Mediation analyses were performed by utilizing the quasi-Bayesian Monte Carlo approximation with 10,000 simulations. The proportion mediated reported is calculated by dividing the causal-mediated effect by the total effect. The mediation analysis included age, sex, education, and depressive symptoms as covariates for the model. In a second step, we included physical activity as an additional covariate.

## Results

### Characteristics of the sample

The main sample was comprised of 142 participants who had been clinically evaluated close to the time of death (median [interquartile range (IQR)], 0.67 [0.43–0.97] years). Ninety-eight of the participants (69%) were female and the median (IQR) age at death was 89.21 (85.4–92.6) years. Sixty-three participants (44.4%) were NCI, 47 (33.1%) were diagnosed with MCI, and 32 (22.5%) were diagnosed with AD dementia. Sample characteristics and comparisons across diagnostic groups are reported in Table 1. In comparison to the AD dementia group, the NCI group had higher social activity (median [IQR], 2.33 [1.75–2.67] vs 1.67 [1.33–2.17]; *p* = 0.004; Fig. S2a), better global cognition (0.03 [−0.27–0.30] vs −1.64 [−2.17 to −1.15]; *p* < 0.0001; Fig. S2b), lower LC tangle density (0.96 [0.56–1.46] vs 2.57 [0.68–4.38]; *p* < 0.0001; Fig. S2d), lower NFT burden (0.16 [0.07–0.33] vs 0.44 [0.25–1.08]; *p* = 0.0006; Fig. S2e), and lower Aβ burden (1.15 [0.06–3.58] vs 4.12 [2.26–7.87]; *p* = 0.0004; Fig. S2f). The NCI group also had better global cognition than the MCI group (0.03 [−0.27–0.30] vs −0.61 [−0.81–0.26]; *p* < 0.0001; Fig. S2b) as well as lower LC tangle density (0.96 [0.56–1.46] vs 1.72 [0.95–2.84]; *p* = 0.05; Fig. S2d), lower NFT burden (0.16 [0.07–0.33] vs 0.36 [0.20–0.72]; *p* = 0.05; Fig. S2e), and lower Aβ burden (1.15 [0.06–3.58] vs 2.97 [1.13–7.96]; *p* = 0.003; Fi g. S2f). There were no significant differences in LC neuronal density, number of APOE ε4 carriers, hours of physical activity (Fig S2c), or depressive symptoms between diagnostic groups.

**Table 1:** Demographic and clinical data.

Characteristic	Median [IQR]				Group comparisons
	Overall (*N* = 142)	NCI (*n* = 63)	MCI (*n* = 47)	AD dementia (*n* = 32)	
Age at death (years)	89.21 [85.42–92.60]	88.22 [84.17–90.63]	89.50 [87.06–92.62]	92.35 [88.28–93.68]	NCI < AD (*p* = 0.0006)
Women, No. (%)	98 (69%)	47 (75%)	32 (68%)	19 (59%)	ns
Education (years)	14.00 [12.00–16.00]	14.00 [12.00–16.00]	15.00 [12.00–16.00]	16.00 [13.75–18.00]	NCI < AD (*p* = 0.002)
Depressive symptoms	1.00 [0–3.00]	1.00 [0–3.00]	1.0 [0–3.00]	2.00 [1.00–3.25]	ns
APOE ε4 carrier	24 (16.90%)	7 (11%)	11 (23%)	6 (19%)	ns
Social activity	2.00 [1.67–2.67]	2.33 [1.75–2.67]	2.00 [1.67–2.42]	1.67 [1.33–2.17]	NCI > AD (*p* = 0.004)
Cortical Aβ burden	2.57 [0.20–5.45]	1.15 [0.06–3.58]	2.97 [1.13–7.96]	4.12 [2.26–7.87]	NCI < MCI (*p* = 0.003) NCI < AD *(p* = 0.0004)
Cortical NFT burden	0.26 [0.10–0.58]	0.16 [0.07–0.33]	0.36 [0.20–0.72]	0.44 [0.25–1.08]	NCI < MCI *(p* = 0.05) NCI < AD (*p* = 0.0006)
LC tangle density	1.32 [0.62–2.61]	0.96 [0.56–1.46]	1.72 [0.95–2.84]	2.57 [0.68–4.38]	NCI < AD (*p* = 0.0001) MCI < AD (*p* = 0.05)
LC neuronal density	44.65 [31.94–57.39]	44.80 [37.46–54.84]	47.97 [31.06–60.63]	40.50 [26.17–53.55]	ns
Physical activity^a^	1.17 [0.00–3.50]	1.25 [0.00–3.50]	1.88 [0.00–3.69]	0.10 [0.00–1.34]	ns
Global cognition	−0.47 [−0.93–0.02]	0.03 [−0.27–0.30]	−0.61 [−0.81 to −0.26]	−1.64 [−2.17 to −1.15]	NCI > MCI (*p* < 0.0001) NCI > AD (*p* < 0.0001) MCI > AD (*p* < 0.0001)
Post-mortem interval (hours)	5.93 [4.83–8.28]	5.67 [4.83–7.60]	6.55 [5.25–10.18]	5.66 [4.72–7.85]	NCI < MCI (*p* = 0.02)
Time between clinical visit and death (years)	0.68 [0.43–0.97]	0.83 [0.48–1.17]	0.70 [0.43–0.90]	0.50 [0.32–0.79]	ns

Social activity, physical activity, and cognition measurements were closest to the time of death.

*Aβ* Amyloid-beta, *AD* Alzheimer’s disease, *APOE* Apolipoprotein E, *NCI* no cognitive impairment, *LC* locus coeruleus, *MCI* mild cognitive impairment, *NFT* neurofibrillary tangles.

^a^*n* = 1 missing data.

Most participants in the main sample also had cognitive and activity data available from their first clinical visit (median [IQR], 5.41 [3.85–6.77] years prior to death; Table S1). Demographics and pathologic burden of this sample (*n* = 126) were similar to the main sample (Table S1). However, as expected, first global cognition scores were overall higher compared to cognitive scores close to the time of death (*t* = −5.35, *p* < 0.001), as were first compared to later social activity and physical activity scores (social activity: *t* = −5.73, *p* < 0.001; physical activity: *t* = −2.53, *p* = 0.012).

### Social activity analyses

**Fig. 1:**
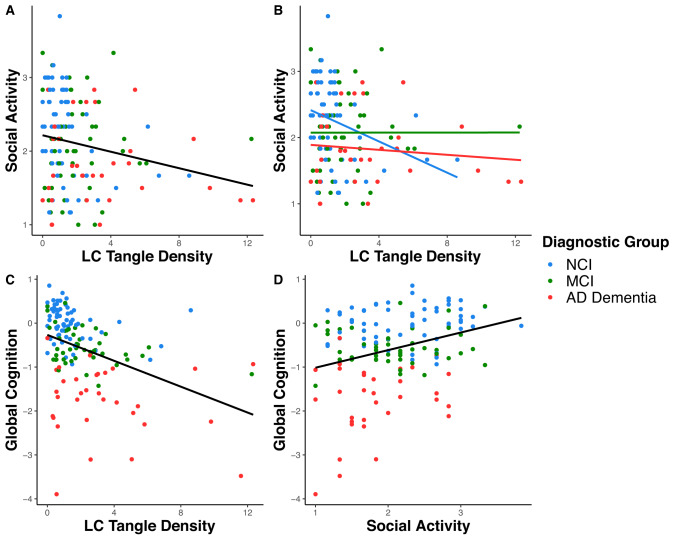
Associations of social activity, Locus Coeruleus tangle density, and cognition by diagnostic group. **A** Association between LC tangle density and social activity, with a trend line fit to the full sample. **B** Association between LC tangle density and social activity, with individual trend lines fit to the no cognitive impairment (NCI), mild cognitive impairment (MCI), and AD dementia groups. Associations between LC tangle density (**C**) and social activity (**D**) with global cognition, with trend lines fit to the full sample. Social activity and cognition measurements were closest to the time of death.

In the first series of models, lower LC tangle density was associated with greater social activity, adjusting for age, sex, and education (Table 2, model 1a: $\beta$=−0.05; *t* = −2.30; *p* = 0.023; Fig. 1a). In analogous models, neither LC neuronal density, Aβ, NFT, nor depressive symptoms were associated with social activity when controlling for the same covariates (Table S2 models S2.1–4). LC tangle density remained associated with social activity when further controlling for depressive symptoms, Aβ, NFT, and LC neuronal density in a combined model (Table 2, model 1b: $\beta$=−0.06; *t* = −2.15; *p* = 0.033). Findings were similar when controlling for each covariate separately (Table S3, models S3.1–4). In secondary models, LC tangle density was also associated with social activity at the first clinical visit, controlling for age, sex, and education ($\beta$ = −0.05; *t* = −2.23; *p* = 0.028; *n* = 126; Table S4) and for all covariates ($\beta$=−0.06; *t* = −2.24; *p* = 0.027; Table S5). Physical activity was not related to LC tangle density or social activity (Fig. S3b, d).

**Table 2:** Associations of social activity with LC tangle density.

Model	β Estimate (95% CI)	Test statistic	*P*-value
Model 1a			
Age	−0.02 (−0.04–0.00)	1.94	0.054
Sex (reference: female)	0.09 (−0.13–0.32)	0.81	0.419
Education	0.03 (−0.01–0.07)	1.28	0.203
LC tangle density	−0.05 (−0.09 to −0.01)	−2.3	**0.023**
Model 1b			
Age at death	−0.02 (−0.04 to −0.00)	−2.07	0.040
Education	0.03 (−0.01–0.07)	1.30	0.195
Sex (reference: female)	0.12 (−0.11–0.35)	1.00	0.319
Depressive symptoms	−0.03 (−0.08–0.02)	−1.17	0.244
Cortical Aβ burden	−0.06 (−0.16–0.03)	−1.32	0.190
Cortical NFT burden	0.15 (−0.07–0.37)	1.35	0.179
LC neuronal density	0.00 (−0.00–0.01)	0.28	0.777
LC tangle density	−0.06 (−0.11 to −0.00)	−2.15	0.033
Model 2: between groups			
Age	−0.01 (−0.03–0.01)	−1.06	0.289
Sex (reference: female)	0.11 (−0.11–0.34)	0.99	0.323
Education	0.04 (−0.00–0.08)	1.84	0.067
LC tangle density × MCI	0.11 (−0.01–0.24)	1.75	0.082
LC tangle density × AD dementia	0.08 (−0.03–0.20)	1.43	0.156
Model 3a: within NCI Group			
Age	−0.01 (−0.04–0.01)	−1.14	0.26
Sex (reference: female)	−0.03 (−0.41–0.34)	−0.17	0.867
Education	0.05 (−0.01–0.11)	1.52	0.133
LC tangle density	−0.1 (−0.20 to −0.01)	−2.14	**0.036**
Model 3b: within CI Group (Combined MCI & AD Dementia Groups)			
Age	−0.01 (−0.04–0.01)	−0.97	0.335
Sex	0.18 (−0.11–0.47)	1.22	0.227
Education	0.02 (−0.03–0.08)	0.78	0.438
LC tangle density	−0.02 (−0.07–0.03)	−0.81	0.422

Bold values indicate statistical significance *p* < 0.05.

Social activity measurement was closest to the time of death.

*Aβ* Amyloid-beta, *AD* Alzheimer’s disease, *CI* cognitively impaired, *NCI* no cognitive impairment, *LC* locus coeruleus, *MCI* mild cognitive impairment, *NFT* neurofibrillary tangles.

In the second series of models, the interaction of diagnostic group and LC tangle density was not significantly associated with social activity, adjusting for age, sex, and education, using the NCI group as reference (Table 2, model 2). However, visualization of estimated differences across diagnostic groups from this model suggested a negative association of LC tangle density and social activity for the NCI group (Fig. 1b). When examining the NCI group alone, lower LC tangle density was significantly associated with higher social activity, controlling for age, sex, and education (Table 2, model 3a: $\beta$ = −0.10; *t* = −2.14; *p* = 0.036). In the CI group (combined MCI and AD dementia groups), this same association was not significant (Table 2, model 3b).

### Cognition analyses

In the full sample, lower LC tangle density was associated with better cognitive performance adjusting for age, sex, education, and depressive symptoms (Table 3, model 4a: $\beta$ = −0.13; *t* = −4.63; *p* < 0.001; Fig. 1c). Higher social activity close to death was also associated with better cognitive performance, adjusting for the same covariates (Table 3, model 4b: $\beta$ = 0.35; *t* = 3.13; *p* = 0.002; Fig. 1d). This association was consistent for social activity at the first clinical visit (first social activity: $\beta$ = 0.35; *t* = 3.09; *p* = 0.003; *n* = 126). Physical activity was not related to cognition (Fig. S3c).

**Table 3:** Linear models for associations of cognition with locus coeruleus tangle density and social activity.

Model	β estimate (95% CI)	Test statistic	*P*-value
Model 4a			
Age	−0.03 (−0.05 to −0.01)	−2.45	**0.016**
Sex (reference: female)	−0.04 (−0.33–0.25)	−0.28	0.78
Education	−0.03 (−0.08–0.03)	−1.00	0.319
Depressive symptoms	−0.08 (−0.14 to −0.02)	−2.53	**0.012**
LC Tangle Density	−0.13 (−0.18 to −0.07)	−4.63	**<0.001**
Model 4b			
Age	−0.03 (−0.05 to −0.01)	−2.50	**0.013**
Sex (reference: female)	−0.08 (−0.38–0.22)	−0.50	0.616
Education	−0.04 (−0.09–0.01)	−1.55	0.124
Depressive symptoms	−0.07 (−0.14 to −0.01)	−2.24	**0.027**
Social activity	0.35 (0.13–0.57)	3.13	**0.002**

Bold values indicate statistical significance *p* < 0.05.

Social activity measurement was closest to the time of death.

*Aβ* Amyloid-beta, *LC* locus coeruleus.

**Fig. 2:**
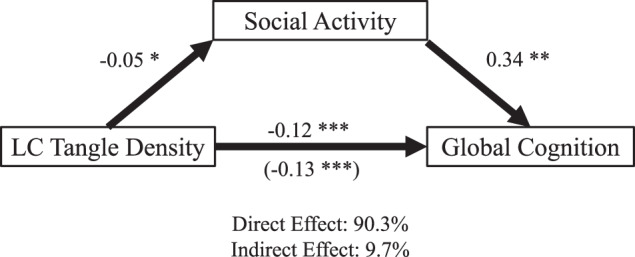
Mediation by social activity on the association of LC tangle density and global cognition. Analyses for social activity as a mediator of the association between locus coeruleus tangle density and global cognition (*n* = 142; mediation effect [ACME]: *p* = 0.023). *p < 0.05, **p < 0.001, ***p < 0.0001. Social activity and cognition measurements were closest to the time of death.

A mediation model was performed to clarify the relationship between social activity, LC tangle density, and cognition. This revealed that the negative association between LC tangle density and cognitive performance was partially mediated by social activity adjusting for age, sex, education, and depressive symptoms (mediation effect: $\beta$ = −0.13, *p* < 0.0001, 95% CI [−0.20 to −0.07]; proportion mediated: $\beta$ = 0.10, *p* = 0.02, 95% CI [0.008–0.3], *n* = 142; percent direct effect: 90.3%; percent indirect effect: 9.7%; Fig. 2). Findings remained the same when adjusting for physical activity (Fig. S4) or when considering first social activity rather than social activity close to death as the mediator (Fig. S5). In the alternative model, first cognitive performance was not a mediator between LC tangle density and social activity close to death (Fig. S6).

## Discussion

In this clinical-pathologic study of NCI and CI older adults, we investigated whether LC tangle pathology was associated with levels of antemortem social activity and cognitive performance and the specificity of these associations. First, we found that hyperphosphorylated tau aggregation in the LC was associated with lower levels of social activity in the sample and specifically in the NCI group. Moreover, this association was independent of cortical Aβ and NFT burden. This provides new evidence that the LC–NA system is involved in late-life social activity in older adults, and further, that social activity may be impacted by local brainstem pathology prior to, or independent of, cortical AD pathology. Second, we found that LC tangle pathology was also associated with lower cognitive performance, and this association was partially mediated by level of social activity, measured earlier in life or closer to death. This suggests that a component of cognitive resilience or cognitive vulnerability to LC tangle pathology is explained by level of social activity, with greater social activity favoring better cognitive performance.

The LC–NA system is known to play a critical role in the regulation of arousal and state-dependent cognitive processes in humans, but this system has not been directly implicated in social function. However, the complex anatomic and functional organization of LC–NA system in animals and humans are subjects of ongoing research and the full scope of its corresponding effects on cognitive and behavioral flexibility in humans are not yet understood.

LC–NA neurons originate in small bilateral nuclei adjacent to the fourth ventricle in the pontine brainstem [7]. These NA neurons arborize and project extensively throughout the central nervous system, particularly to the cerebral hemispheres and other forebrain regions [7]. Important areas of projection include the prefrontal cortex involved in selective attention, working memory, and sensory function, and also to the hippocampus, amygdala, and hypothalamus, involved in memory, emotion and stress responses [32]. Neuroanatomic studies indicate that LC–NA axonal projections are modular and target-specific, thus enabling differential modulation of cognition and behavior in response to varying environmental contingencies [8]. In turn, LC–NA neurons receive afferent input from many regions of the forebrain and brainstem, as well as local input from GABAergic neurons in the peri-LC region [8]. Through this widespread afferent and efferent network, the LC–NA system modulates attention, sensory perception, working memory and memory retrieval, and long-term memory formation, most notably, in response to conditions of novelty, changes in reward contingencies, or emotional arousal [8]. Thus, the LC–NA system has been described as “a critical component of the neural architecture supporting interaction with, and navigation through, a complex world” [33].

The findings from this study build on our own prior research using MAP data which showed that LC tangle pathology was associated with lower episodic memory scores in the full sample, and also in the NCI subsample, adjusting for cortical Aβ [6]. Here we show that the association of LC tangles with lower cognitive performance is independent of both cortical Aβ and NFT. Further, there is an analogous association of LC tangle pathology with lower social activity that also appears to be present, specifically, in the NCI group. Though interpretations are limited by the observational and cross-sectional design of this study, these data suggest that LC tangle accumulation may impact LC–NA network function to degrade cognition and social function prior to or independent of cerebral AD pathology and prior to the onset of cognitive impairment.

In a broader context, these findings align with a new consensus research framework which identifies the LC and other subcortical neuromodulatory systems as initial sites of AD pathogenesis and other primordial neurodegenerative processes [13, 34]. This framework recognizes LC tangle pathology as a potential biomarker of early cognitive and behavioral change and as a strategic signal of AD risk prior to cerebral Aβ and NFT involvement (13, 29). Thus, recognition of neurobehavioral symptoms, such as social declines, and monitoring of LC integrity and neurochemical function could enhance early identification of individuals at increased risk of developing AD and inform the development of new prevention trials.

A novel and key finding from our study is that the association of LC tangle pathology with poorer cognitive performance is partially mediated by level of social activity such that greater social activity favors better cognitive performance. Notably, cognitive performance did not mediate the relationship between LC tangle pathology and social activity, further supporting the hypothesized role of social activity as a mediator of LC-related cognitive processes rather than the converse. Moreover, this relationship was also independent of level of physical activity, a putative component of cognitive reserve that was not related to either LC tangle density or social activity in these data.

Mechanisms by which social activity mediates LC-related cognitive processes are unknown. One possible explanation is that cognitive and social activities utilize common or overlapping LC–NA neural circuits with greater social activity increasing the plasticity and strength of these common circuits [13, 35]. Novel and dynamic aspects of social activity may be most effective at eliciting noradrenergic activation and neuroplasticity, as noted for other forms of environmental enrichment [14]. Social activity may also build capacity to engage accessory neural circuits to compensate for pathology during cognitive tasks.

A longstanding pattern of high social engagement may also impact brain health by mitigating physiologic stress responses and allostatic load. Among older adult participants in the Health and Retirement Study, greater family, friend, and group contacts assessed over multiple waves were associated with lower systolic blood pressure and of plasma C-reactive protein eight years later [36]. Levels of social support and social strain showed different patterns of biomarker associations, (higher social support with lower systolic blood pressure and waist circumference; lower social strain with lower waist circumference and body mass index) suggesting independent and shared effects of these social connection domains on health [36]. While social relationships and their health implications are studied according to separable domains of “structure” (numbers of ties or interactions), “function” (benefits supplied) and “quality” (e.g., sense of belonging, relationship strain), the extent of social interactions can be a foundation upon which the other domains operate [37]. In humans, chronic perceived social isolation (loneliness) activates a conserved transcriptional response to adversity marked by increased expression of pro-inflammatory genes and decreased anti-viral and antibody responses [38]. This HPA-axis and sympathetic nervous system-mediated response could be an indirect pathway by which the extent and adequacy of social relationships affect levels of neuroinflammation, systemic inflammation, and brain health.

There are limitations to this study. Interpretations of the causal relationships of LC tangle pathology, social activity and cognition are limited by the cross-sectional design of the analyses. Therefore, these models were built based on biologically plausible hypotheses, informed by the cognitive reserve literature, and cannot test all directionalities [39]. Moreover, certain interpretations rely on an assumption that the measure of social activity close to time of death is representative of a longer pattern of activity. Our secondary analyses are consistent with this assumption as they revealed that social activity measured approximately five years prior to death replicated our key findings. Our analyses controlled for depressive symptoms but no other neuropsychiatric symptoms which have been associated with tau pathology in early AD and could contribute to social activity level [40, 41]. In addition, older individuals with higher cognitive reserve exhibited a more intact locus coeruleus in previous in vivo studies [6, 42, 43]. Unfortunately, the MAP-study does not collect information on premorbid IQ. It would be valuable for future studies to examine the links between premorbid IQ and other socio-behavioral proxies of cognitive reserve in older adults [39]. Finally, studies examining neuronal density in the LC have reported wide variability possibly related to the methodology, sample size, disease stages and whether only TH-positive or neuromelanin bearing neurons were included [44–51]. While very labor-intense, a comprehensive, unbiased stereological evaluation is required to better understand the evolution of LC neuronal count as a function of tangle accumulation and disease progression.

In conclusion, we found that hyperphosphorylated tangle pathology in the LC was associated with lower levels of antemortem social activity in older adults, and specifically among those who were cognitively unimpaired. This association partially mediated the association of LC tangle pathology and cognition. These findings suggest that early pathological accumulations in the brainstem may impact social function, and this may, in turn, influence cognitive performance more broadly. These findings add to a growing body of evidence that pathological changes in the LC have measurable clinical correlates and that preserved structure and function of the LC could be a marker of cognitive reserve.

## Supplementary information

Supplemental Material

## Data Availability

Data from ROSMAP is available upon request at https://www.radc.rush.edu.
