# Supplementary material for: Social activity mediates locus coeruleus tangle-related cognition in older adults
Source: Mol Psychiatry. 2024 Feb 15;29(7):2001–8. doi: 10.1038/s41380-024-02467-y (PMC11408254; doi:10.1038/s41380-024-02467-y)
Supplement: Supplementary file 1 — Supplemental Material [file 41380_2024_2467_MOESM1_ESM.docx]

**Supplementary Online Content**

Benjamin S. Zide, MSt, Nancy J. Donovan, MD, Soyoung Lee, MD, Sukriti Nag, MD, PhD, David A. Bennett, MD, Heidi I. L. Jacobs, PhD. Social activity mediates locus coeruleus tangle-related cognition in older adults.

**Methods.** Additional details on the neuropathologic measures.

**Figure S1.** Micrograph of locus coeruleus and frontal cortex neuropathology.

**Table S1.** Demographics and clinical data of the sample at the first clinical visit.

**Figure S2.** Comparisons of social activity, global cognition, physical activity, LC tangle density, and AD pathological markers across diagnostic groups in the entire sample.

**Figure S3.** Associations of physical activity, locus coeruleus tangle density, cognition and social activity.

**Table S2.** Associations of social activity with individual predictors.

**Table S3.** Associations of social activity with LC tangle density controlling for various covariates.

**Table S4.** Associations of social activity at the first clinical visit with LC tangle density.

**Table S5.** Associations of social activity at the first clinical visit with LC tangle density**,** adjusting for all covariates.

**Figure S4.** Mediation by social activity on the association of LC tangle density and global cognition, with additional adjustment for physical activity.

**Figure S5.** Mediation by social activity at the first clinical visit on the association of LC tangle density and global cognition.

**Figure S6.** Mediation by global cognitive performance at the first clinical visit on the association of LC tangle density and social activity.

This supplementary material has been provided by the authors to give readers additional information about their work.

**Methods.** Additional details on the neuropathologic measures.

*Locus Coeruleus (LC) Neuron Density*: Neurons were detected in 20 µm sections with an antibody to tyrosine hydroxylase (1:750; Immunostar, Hudson, WI). Immunohistochemistry was performed using an indirect immunoenzyme horseradish peroxidase method with 3,3′diamino-benzidine as the chromogen in an autostainer (Leica Bond Max; Leica Microsystems) using a kit (Bond Polymer Refine Detection Kit; Leica Microsystems). Nickel chloride (5%) added to the 3, 3′diaminobenzidine substrate (Vector Laboratories, Burlingame, CA) enhanced the signal of the tyrosine-hydroxylase positive neurons and their processes to a brownish black color (Fig S-A & B). The area of the LC was outlined using software available on the Stereo Investigator Program (MBF Biosciences, Williston, VT) attached to an Olympus BX60 microscope equipped with a motorized stage. All neurons within the outline were quantified manually at x 400 magnification and divided by the area of the LC to obtain the neuron density/mm^2^. Data from the four sections containing the LC were averaged to obtain a mean neuron density/mm^2^, the primary measure used in these analyses. The inter-rater reliability of these neuronal density measures in the LC, estimated in a subset of 12 participants, was high (r = 0.97).

*Locus Coeruleus NFT*: Neurofibrillary tangles were detected in 20 µm sections using an anti-paired helical filament antibody specific for phosphorylated tau (AT8, Innogenetics, San Ramon, CA, 1:1000). Since this antibody detects both pretangles and tangles, all neurons containing focal non-fibrillar intracytoplasmic brown immunostaining (pretangles) or diffuse fibrillar intracytoplasmic immunostaining with extension to a proximal dendrite (tangles) were included in the counts (Fig S1-C). The outline of the LC obtained previously was superimposed on the AT8 immunostained sections and all tangles within this area were quantified manually and results were expressed as LC tangle density/mm^2^.

*Brain AD Neuropathological Markers*

*Brain NFT/mm^2^:* Manual counts of NFT were done in a 1 mm^2^ area having the highest density of these structures using 6 µm sections from five brain regions stained by the modified Bielschowsky silver stain (Fig. S1-D) at a total magnification of x100. Counts of each region were scaled by dividing by the corresponding standard deviation and then averaging the 5 scaled regional measures to obtain a summary measure for brain NFT/mm^2^ for use in analyses as described previously^1^. The summary measure for NFT/mm^2^ was computed only if data was available for at least 2 of the 5 regions.

*Brain Aβ burden:* The percentage area occupied by Aβ plaques in eight brain regions was obtained using two 20 µm sections for each of the six neocortical regions and 20 µm sections of the hippocampus obtained from serial 1 cm slabs of the entire hippocampus. Video images of Aβ immunostaining were captured using a systematic random sampling scheme with a custom algorithm^2^. A region of interest was outlined at low power with Stereo Investigator software V.5 and an Olympus BX51 microscope. A grid of predetermined size was then randomly placed by the software program over the outlined area so that 20–50% of the region was sampled. After camera and illumination calibration, the objective was increased to x 20, and at each sampling site, 24‐bit images were obtained with an automatically positioned motorized stage. In each region, the percentage of area occupied by Aβ immunoreactive pixels was calculated using the custom algorithm^2^ and the regional indices were averaged to provide a composite index of amyloid burden, as described in previous research^3^.

**Figure S1.** Micrograph of locus coeruleus and frontal cortex neuropathology.

**
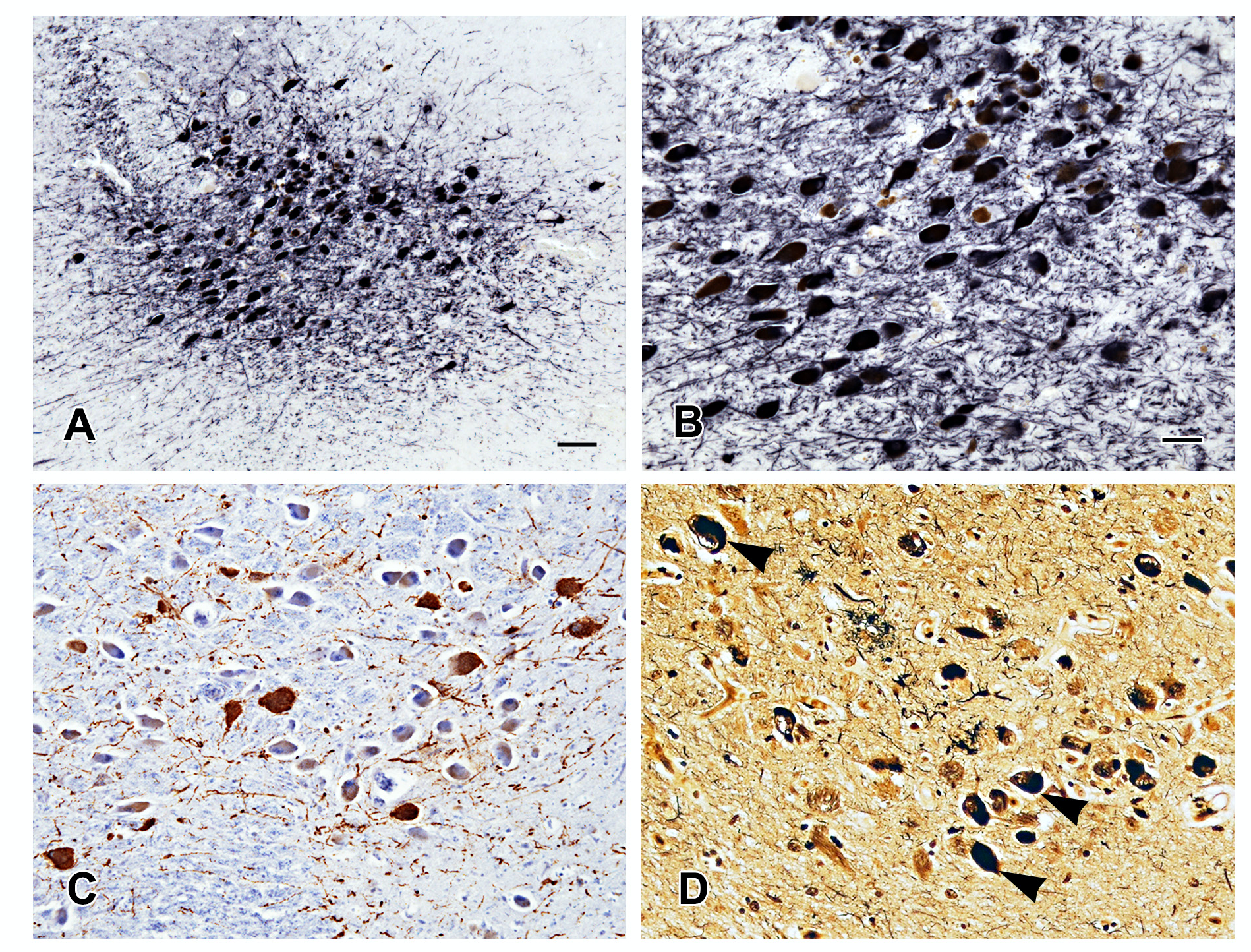
**

Note: Locus coeruleus (A-C) and frontal cortex (D). A) Low power micrograph of the left LC showing tyrosine hydroxylase positivity which facilitates outlining the area for quantitation. B) Higher magnification of tyrosine hydroxylase positive neurons and processes in the LC. C) LC showing AT8 positive (brown) intracytoplasmic tangles in neurons and neurites. D) Modified Bielschowsky stain showing neurofibrillary tangles (arrowheads) in the frontal cortex neurons. Scale bar = 125 µm (A) and 50 µm (B-D).

**Table S1.** Demographics and clinical data of the sample at the first clinical visit.

| **Characteristic** | **Median [IQR]** | | | |
| --- | --- | --- | --- | --- |
|  | **Overall**  (N=126) | **NCI**  (n=54) | **MCI**  (n=44) | **AD Dementia**  (n=28) |
| Age at Death (years) | 89.21  [85.42-92.60] | 88.16  [84.11-90.50] | 89.57  [87.09-92.73] | 92.35  [88.28-94.81] |
| Women, No. (%) | 88  (69.84%) | 41  (75.93%) | 30  (68.18%) | 17  (60.71%) |
| Education (years) | 14  [12-16] | 14  [12-16] | 14.5  [12-16] | 16  [13.75-18] |
| APOE ε4 carrier | 23  (18.25%) | 6  (11.11%) | 11  (25%) | 6  (21.43%) |
| First social activity | 2.50  [2.17-3.00] | 2.67  [2.33-3.17] | 2.50  [2.33-2.83] | 2.25  [1.83-2.71] |
| Cortical Aβ density | 2.59  [0.42-5.45] | 1.20  [0.05-3.32] | 3.17  [1.23-8.25] | 4.12  [2.12-6.87] |
| Cortical NFT density | 0.27  [0.10-0.58] | 0.13  [0.07-0.29] | 0.38  [0.20-0.79] | 0.44  [0.26-1.04] |
| LC tangle density | 1.26  [0.60-2.58] | 0.90  [0.54-1.43] | 1.70  [0.88-2.77] | 2.57  [0.68-4.40] |
| First physical activity | 2.25  [0.58-3.50] | 1.90  [0.69-2.89] | 2.50  [0.29-4.97] | 2.25  [0.81-4.19] |
| First global cognition | -0.04  [-0.47-0.24] | 0.17  [0.01-0.39] | -0.26  [-0.50-0.12] | -0.57  [-0.94- -0.25] |
| Postmortem interval (hours) | 5.90  [4.83-8.28] | 5.58  [4.83-7.05] | 6.53  [5.25-10.09] | 5.66  [4.72-7.56] |
| Time between past clinical visit and death (years) | 5.41  [3.85-6.77] | 5.58  [4.03-6.54] | 5.41  [3.75-7.13] | 5.08  [3.63-6.13] |

Abbreviations: Aβ: Amyloid-beta; AD: Alzheimer’s disease APOE: Apolipoprotein E; LC: locus coeruleus; MCI: mild cognitive impairment; NCI: no cognitive impairment; NFT: neurofibrillary tangles.

**Figure S2.** Comparisons of social activity, global cognition, physical activity, LC tangle density, and AD pathologic markers across diagnostic groups in the entire sample.


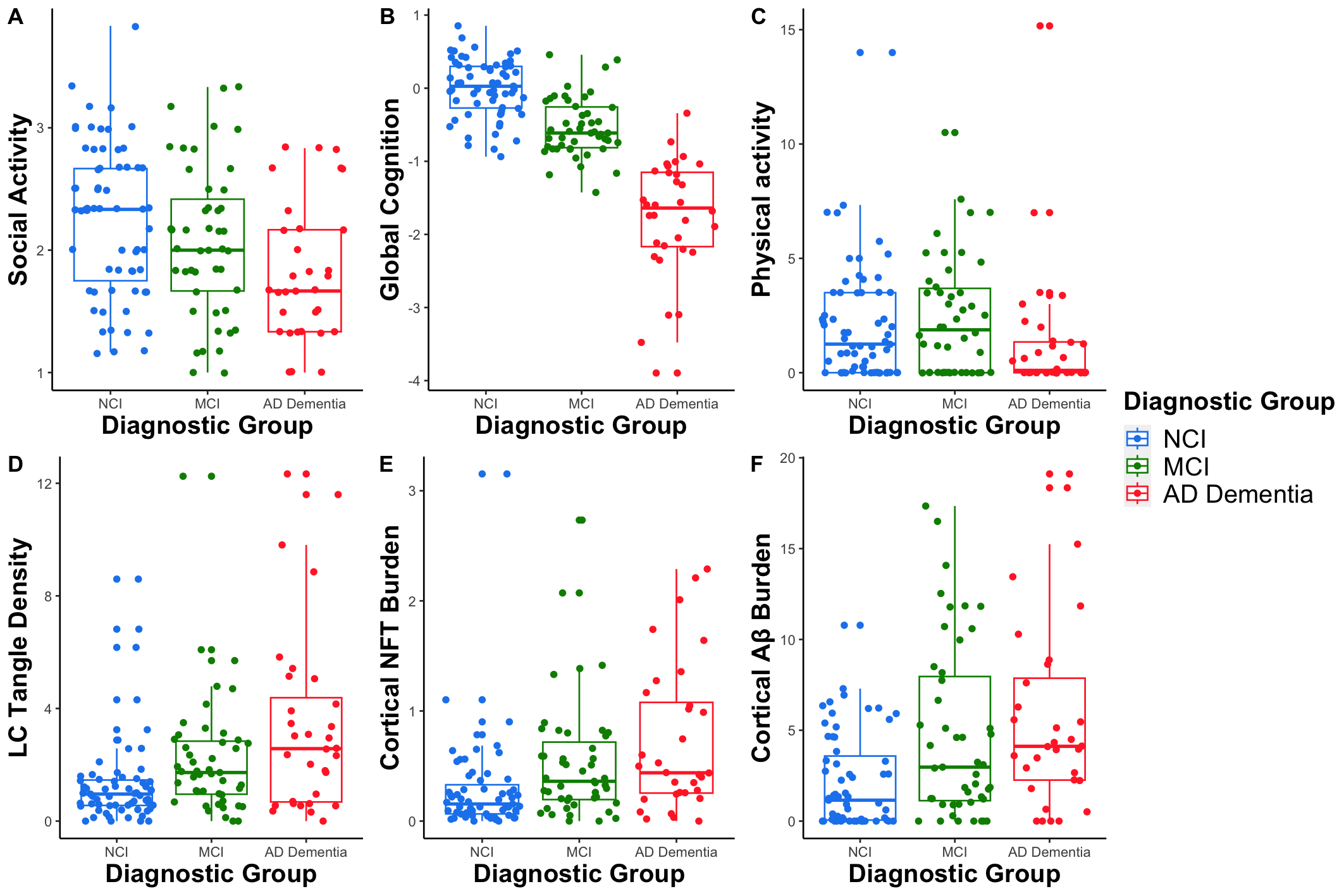


Abbreviations: Aβ: Amyloid-beta; AD: Alzheimer’s disease; LC: locus coeruleus; MCI: mild cognitive impairment; NCI: no cognitive impairment; NFT: neurofibrillary tangles. Cognition and activity data collected closest to death are shown.

**Figure S3.** Associations of physical activity, locus coeruleus tangle density, cognition and social activity.

**
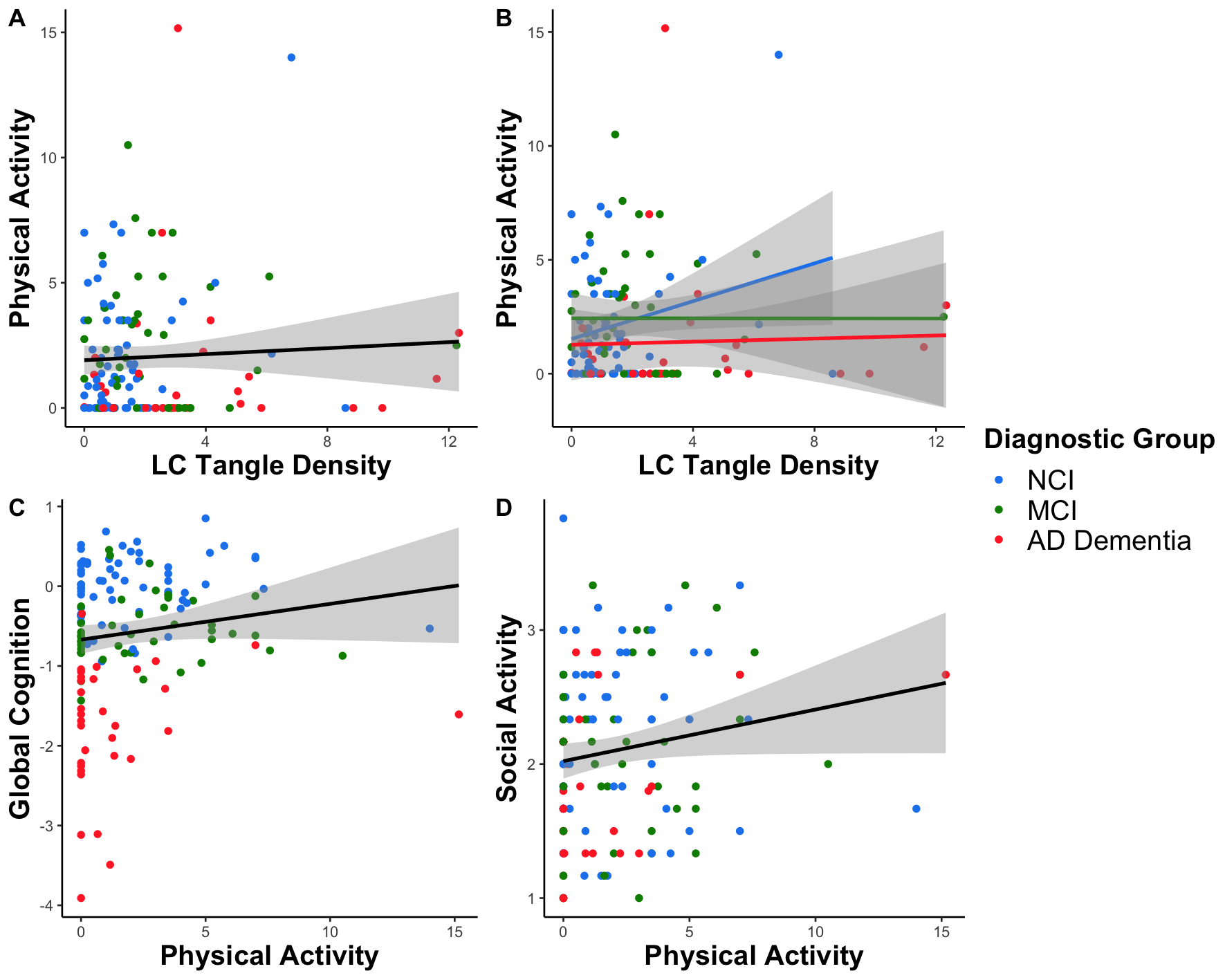
**

Note: A) Physical activity was not related to LC tangle density in the entire group ($\beta$=0.07; t=0.73; *p*=0.47) or B) in the cognitively impaired individuals ($\beta$=-0.01; t=-0.05; *p*=0.96). B) Physical activity was related to LC tangle density in the not cognitively impaired individuals ($\beta$=0.45; t=2.22; *p*=0.030). C) Physical activity was not related to global cognition ($\beta$=0.04; t=1.30; *p*=0.20) or D) social activity $(\beta$=0.03; t=1.30; *p*=0.20). Models were adjusted for age at death, sex, and education. Cognition and activity data collected closest to death are shown.

**Table S2.** Associations of social activity with individual predictors.

**
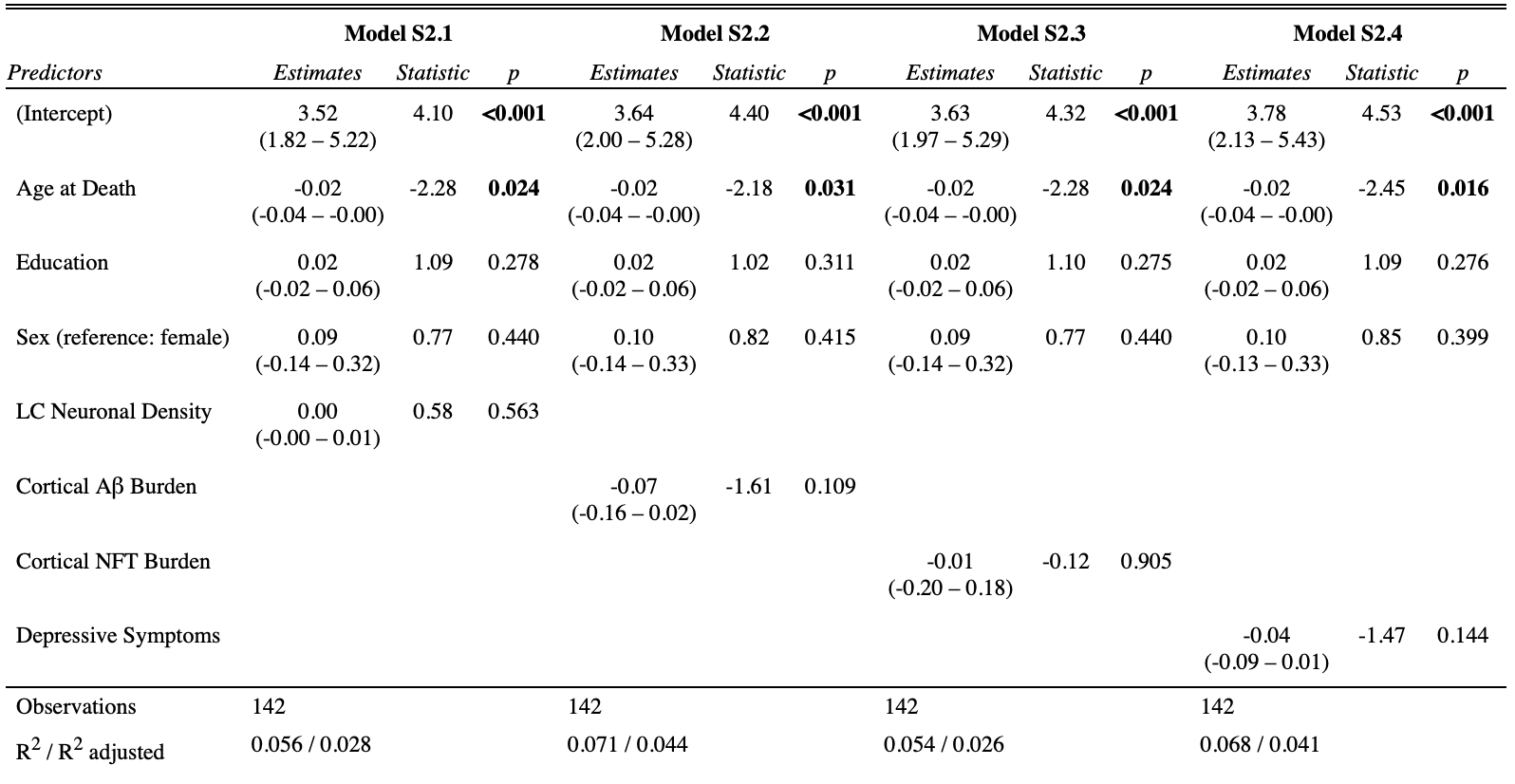
**

Note: Models estimated social activity measures closest to death.

**Table S3.** Associations of social activity with LC tangle density controlling for various covariates.

**
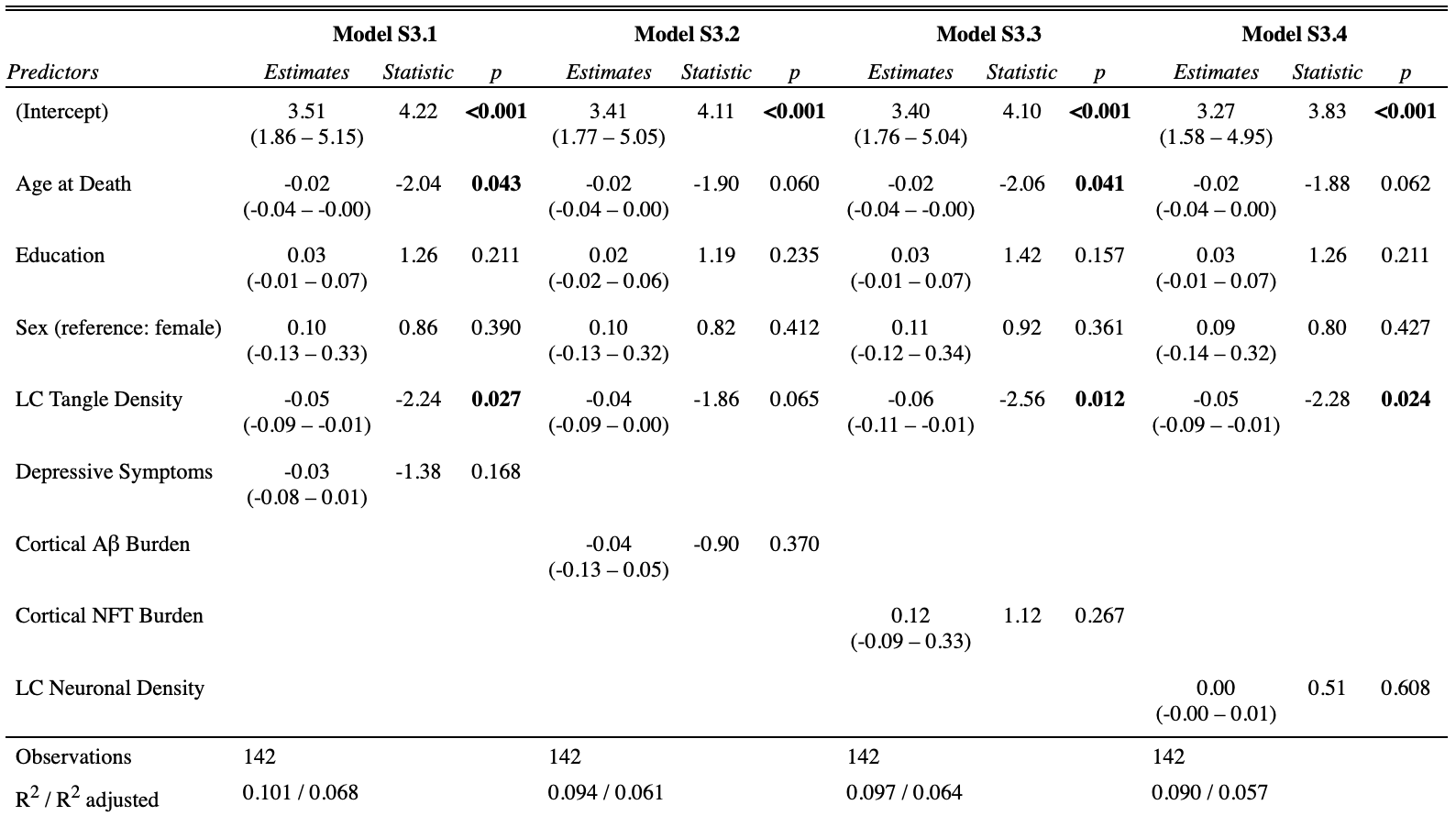
**

Note: Models estimated social activity measures closest to death.

**Table S4.** Associations of social activity at the first clinical visit with LC tangle density**.**


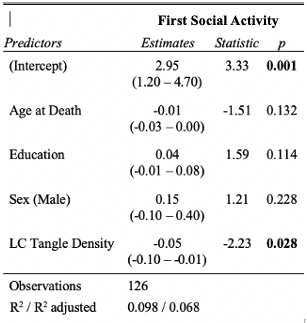


Note: Model estimated social activity data collected at the first clinical visit.

**Table S5.** Associations of social activity at the first clinical visit with LC tangle density**,** adjusting for all covariates.


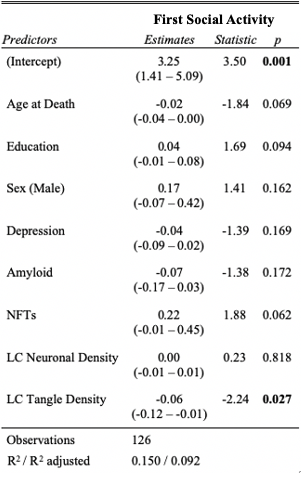


Note: Model estimated social activity data collected at the first clinical visit.

**Figure S4.** Mediation by social activity on the association of LC tangle density and global cognition, with additional adjustment for physical activity.


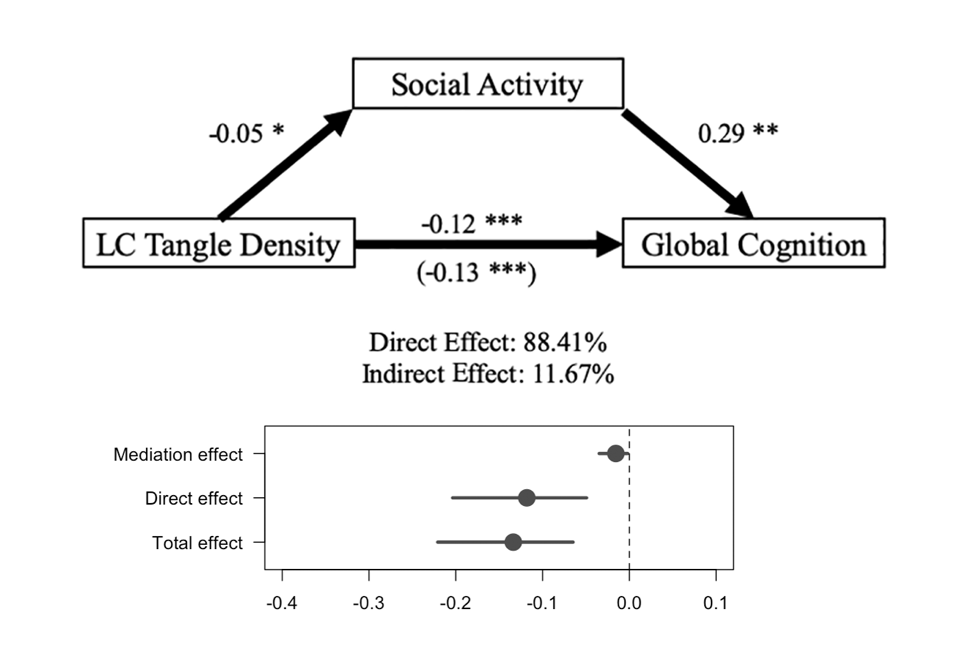


Note: Social activity partially mediates the relationship between LC tangle density and cognitive performance, adjusting for age at death, sex, education, and physical activity (mediation effect: $\beta$=-0.16, *p*=0.016, 95% CI [-0.04 to -0.001]; proportion mediated: $\beta$=0.12, *p*=0.017, 95% CI [0.02 to 0.32], n=141). Cognition and activity data closest to death were used.

**Figure S5.** Mediation by social activity at the first clinical visit on the association of LC tangle density and global cognition.


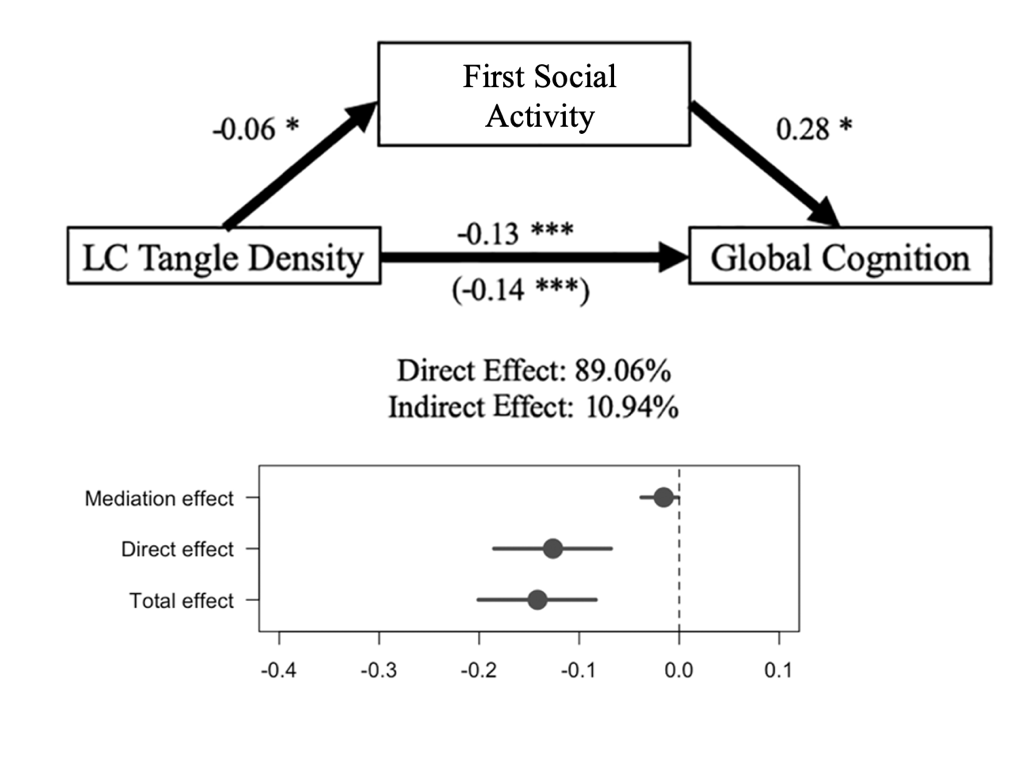


Note: First social activity partially mediates the relationship between LC tangle density and cognitive performance closest to death (adjusting for age, sex and education): mediation effect: $\beta$=-0.15 , *p*=0.031, 95% CI (-0.04 to -0.001); proportion mediated: $\beta$=0.10, *p*=0.03, 95% CI (0.006 to 0.27), n=126. We added past physical activity as an additional covariate, and observed that first social activity remained a significant mediator of the relationship between LC tangle density and cognitive performance closest to death with a direct effect of 89.13% and an indirect effect contribution of 10.87% (mediation effect: $\beta$=-0.15 , *p*=0.037, 95% CI [-0.04 to -0.001]; proportion mediated: $\beta$=0.10 , *p*=0.037, 95% CI [0.005 to 0.28], n=126).

**Figure S6.** Mediation by global cognitive performance at the first clinical visit on the association of LC tangle density and social activity.


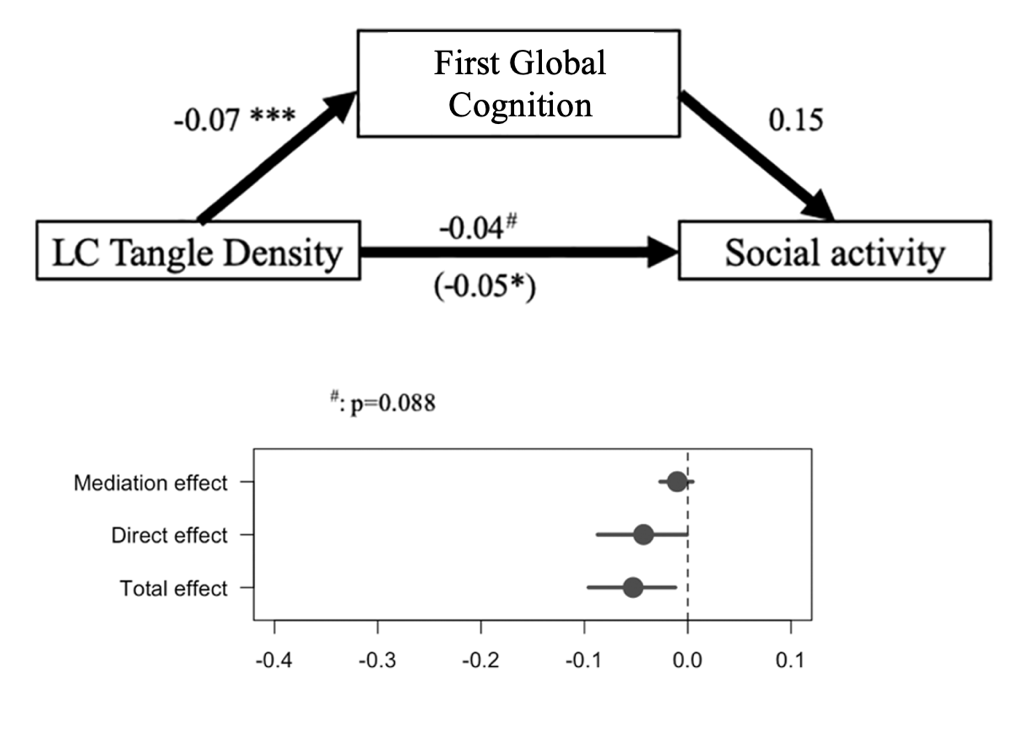


Note: First global cognitive performance did not mediate the relationship between LC tangle density and social activity levels closest to death (mediation effect: $\beta$=-0.10 , *p*=0.168, 95% CI [-0.03 to 0.001]; proportion mediated: $\beta$=0.18 , *p*=0.18, 95% CI [-0.13 to 0.82], n=126). This did not change when adding in physical activity as additional covariate (mediation effect: $\beta$=-0.01, *p*=0.20, 95% CI [-0.03 to 0.01]; proportion mediated: $\beta$=0.16 , *p*=0.21, 95% CI [-0.13 to 0.76], n=126).

**References**

1. Schneider JA, Wilson RS, Bienias JL, Evans DA, Bennett DA. Cerebral infarctions and the likelihood of dementia from Alzheimer disease pathology. *Neurology* 2004; **62**(7)**:** 1148-1155.

2. Mitchell TW, Nissanov J, Han LY, Mufson EJ, Schneider JA, Cochran EJ *et al.* Novel method to quantify neuropil threads in brains from elders with or without cognitive impairment. *J Histochem Cytochem* 2000; **48**(12)**:** 1627-1638.

3. Bennett DA, Schneider JA, Tang Y, Arnold SE, Wilson RS. The effect of social networks on the relation between Alzheimer's disease pathology and level of cognitive function in old people: a longitudinal cohort study. *Lancet Neurol* 2006; **5**(5)**:** 406-412.
